# Biosynthetic potential of the sediment microbial subcommunities of an unexplored karst ecosystem and its ecological implications

**DOI:** 10.1002/mbo3.1407

**Published:** 2024-04-09

**Authors:** Pablo Suárez‐Moo, Alejandra Prieto‐Davó

**Affiliations:** ^1^ Unidad de Química‐Sisal, Facultad de Química Universidad Nacional Autónoma de México Sisal Yucatán México

**Keywords:** karst ecosystem, MAGs, microbial ecology, natural product, secondary metabolite, sediment microbial communities, Yucatan peninsula

## Abstract

Microbial communities from various environments have been studied in the quest for new natural products with a broad range of applications in medicine and biotechnology. We employed an enrichment method and genome mining tools to examine the biosynthetic potential of microbial communities in the sediments of a coastal sinkhole within the karst ecosystem of the Yucatán Peninsula, Mexico. Our investigation led to the detection of 203 biosynthetic gene clusters (BGCs) and 55 secondary metabolites (SMs) within 35 high‐quality metagenome‐assembled genomes (MAGs) derived from these subcommunities. The most abundant types of BGCs were Terpene, Nonribosomal peptide‐synthetase, and Type III polyketide synthase. Some of the in silico identified BGCs and SMs have been previously reported to exhibit biological activities against pathogenic bacteria and fungi. Others could play significant roles in the sinkhole ecosystem, such as iron solubilization and osmotic stress protection. Interestingly, 75% of the BGCs showed no sequence homology with bacterial BGCs previously reported in the MiBIG database. This suggests that the microbial communities in this environment could be an untapped source of genes encoding novel specialized compounds. The majority of the BGCs were identified in pathways found in the genus *Virgibacillus*, followed by *Sporosarcina, Siminovitchia, Rhodococcus*, and *Halomonas*. The latter, along with *Paraclostridium* and *Lysinibacillus*, had the highest number of identified BGC types. This study offers fresh insights into the potential ecological role of SMs from sediment microbial communities in an unexplored environment, underscoring their value as a source of novel natural products.

## Graphical abstract

This study employed an enrichment method and genome mining tools to uncover novel bacterial genomes and explore their biosynthetic potential. The research focused on microbial communities residing in the sediments of an uncharted karst environment, specifically the Polac sinkhole, located on the Yucatán Peninsula.

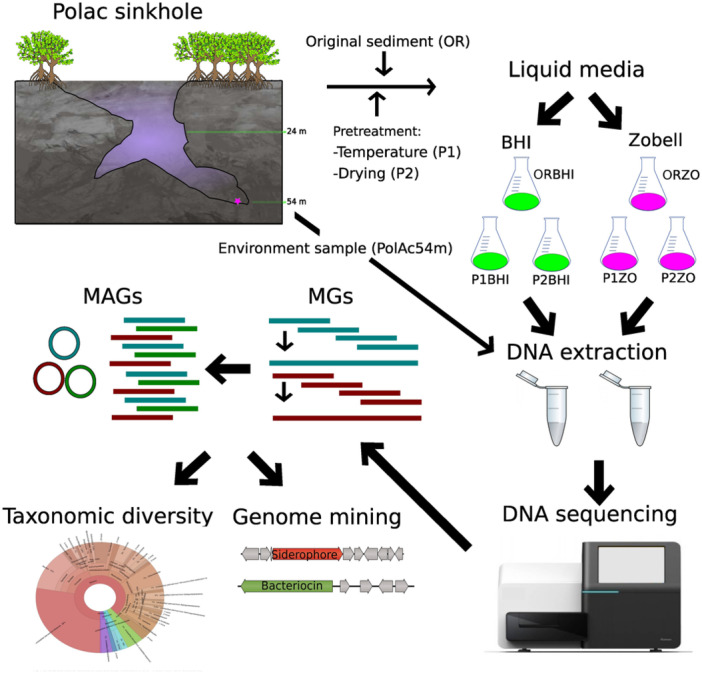

## 1 INTRODUCTION

Multidrug resistance in microbial pathogens is an emerging global challenge threatening future public health (Chen et al., 2019). This critical health threat emphasizes the importance of the discovery of new natural products, whether by using different tools or by exploring new environments (Chen et al., 2019; Li & Rebuffat, 2019; Sekurova et al., 2019). The use of shotgun metagenomic sequencing along with a cultivation‐dependent approach allows taxonomic and functional characterization of a live microbial subcommunity and the reconstruction de novo of near‐complete genomes, termed metagenome‐assembled genomes (MAGs). MAGs are crucial for exploring phylogenies and the ecological and metabolic functions of microorganisms in a specific environment (Chen, Anantharaman, et al., 2020), and they allow genome mining to search for biosynthetic gene clusters (BGCs) (Chen, Chiang, et al., 2021). BGCs are a grouping of all genes responsible for the assembly of secondary metabolites (SMs) (also known as natural products) (Medema et al., 2015), which play roles within their host as defense molecules, virulence factors, or give them survival advantage, additionally, they are of medical and biotechnological importance (Ceniceros et al., 2017; Letzel et al., 2013). To identify BGCs, various computational tools have been developed (Blin et al., 2019; van Heel et al., 2018; Ziemert et al., 2012), and the process is commonly called “genome mining.” BGC identification using genome mining has significantly increased in recent years leading to the exploration of diverse microbial environments such as marine microbial mats (Chen, Wong, et al., 2020), lakes (Chen, Chiang, et al., 2021; Cuadrat et al., 2018), the Arctic Ocean (Rego et al., 2021), marine sediments (Bruce et al., 2022), mangrove sediments (Marfil‐Santana et al., 2021), and marine sponges (Storey et al., 2020). These searches are providing an understanding of microbial ecological and evolutionary processes in these environments and are revealing the potential they have as sources of natural products.

The karst ecosystem of the Yucatán peninsula, Mexico is an underground river ecosystem with unique geological characteristics that represent the only available water resource in the region (Bauer‐Gottwein et al., 2011). This underground ecosystem is exposed above the ground by inland or coastal sinkholes (Schmitter‐Soto et al., 2002). The microbial communities present in this ecosystem can offer a high biotechnological potential as a source of novel molecules for industry (Rojas‐Herrera et al., 2011). However, in this environment, there is still a knowledge gap in terms of taxonomic and functional diversity associated with BGC discovery. The structure of microbial communities in the sinkholes of the peninsula has been studied with culture‐independent approaches using 16S rRNA amplicons (Brankovits et al., 2017; Moore et al., 2020; Stinnesbeck et al., 2018; Suárez‐Moo et al., 2022) and shotgun metagenomics sequencing (Moore et al., 2020). Those previous studies have reported the ecological functions of the microbial communities from the sinkholes, suggesting that their role in the biogeochemical cycles in these ecosystems is important (Brankovits et al., 2017; Suárez‐Moo et al., 2022). Nevertheless, no studies are reporting a search for BGCs. A recent study reported the presence of many multidrug resistance operons, such as the *mtd*ABCD multidrug resistance cluster, and multiple antibiotic resistance *mar* locus genes in the groundwater from the Yucatán aquifer (Moore et al., 2020). The use of a simple computational method to explore the polyketide synthases (PKSs) diversity in well water from the Yucatán aquifer has revealed the biotechnological potential of microbial communities in this overlooked environment as a source of novel and diverse natural products (Marfil‐Santana et al., 2016).

Our previous 16S rRNA amplicon survey of the sediment microbial community in sinkholes of the Yucatán peninsula showed that these ecosystems have particular environmental factors that hold a high microbial diversity, and we hypothesized that the uniqueness of this environment would favor a high BGC diversity. The goal of the present study is to describe the taxonomic and functional diversity associated with BGCs from the sediment microbial communities in a coastal sinkhole of the karst ecosystem in the Yucatán peninsula, using an approach based on culture, shotgun metagenomic sequencing, and genome mining. This study provides evidence that the sinkholes are a rich reservoir of unexplored microbial genomes and metabolic functions, and it demonstrates the ecological and biotechnological importance of sediment microbial communities in this unexplored environment.

## 2 MATERIALS AND METHODS

### 2.1 Sediment samples, cultures, and metagenomic sequencing

**Figure 1:**
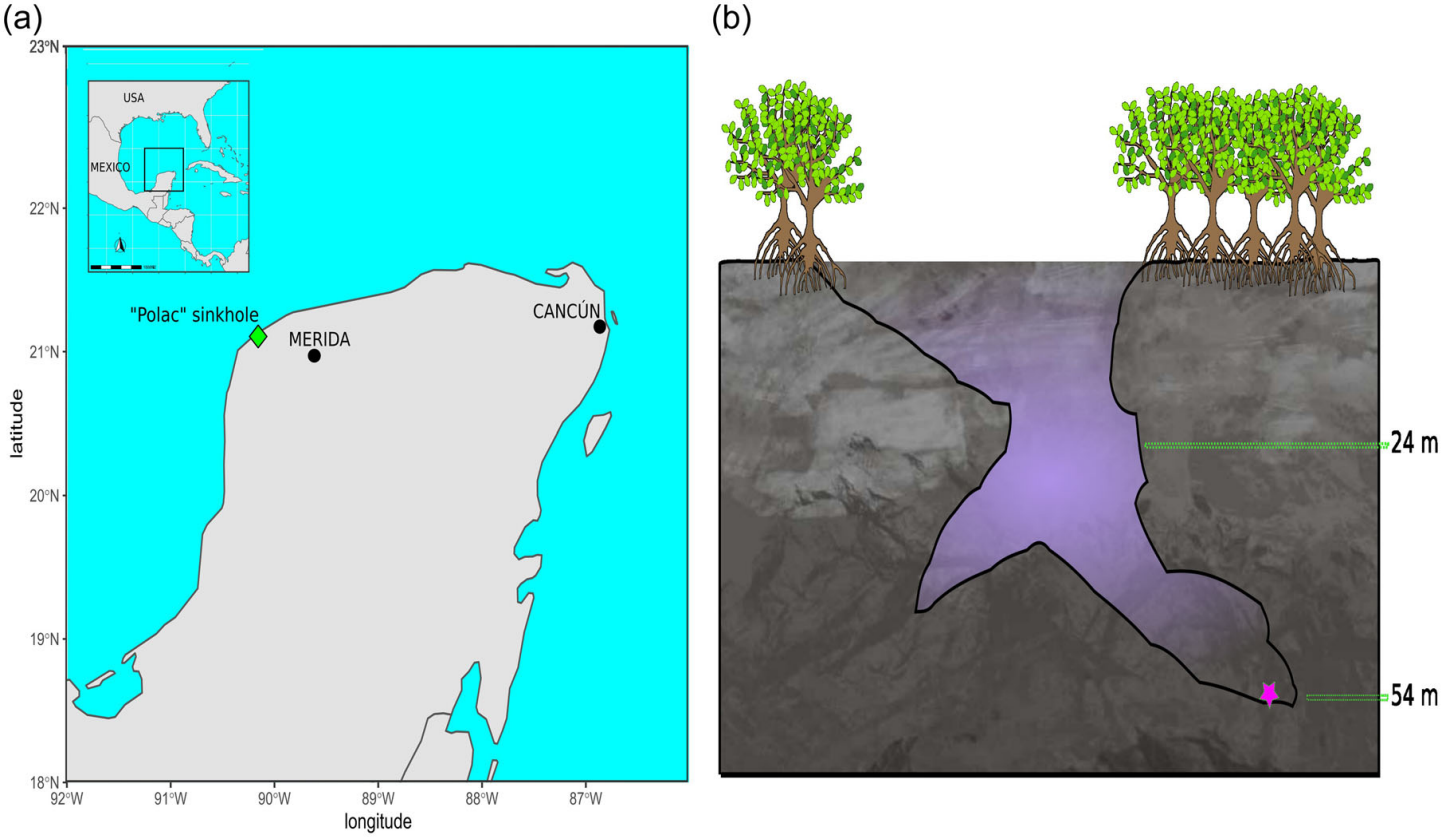
Polac coastal sinkhole. (a) Geographic location (diamond symbol) on the Yucatan Peninsula, Mexico. (b) Profile based on diver's descriptions; sediment zone is indicated with a purple star.

A sediment sample at a depth of 54 m was collected from a coastal sinkhole on the Yucatán peninsula in January 2021 (Figure 1); this sinkhole, Polac, lies within the municipality of Hunucmá, Yucatán, Mexico (21°4′53.8026” N, −90°12′10.5552” W). It is a bottle‐shaped cave with salinity ranging from 21.5 practical salinity units (PSU) in the shallow zone to 39.5 PSU at its deepest zone where it reaches 55 m in depth (Figure 1). The sample was centrifuged to separate water from the sediment and vortexed in sterile sinkhole water to disperse the biofilm covering the sediment particles. Two pretreatments (based on temperature and drying) were developed to reduce the diversity and enhance the functional annotation of the sediment microbial communities (For details, see Supporting Information: Figure A0): (Pretreatment 1) ~100 mg sediment was subjected to a water bath at 55°C for 15 min; and (Pretreatment 2) ~100 mg sediment was dried at room temperature for 12 h under a vertical laminar flux hood. The pretreated sediments and environmental sample (original sediment) (OR) were inoculated into 50 mL of two microbial liquid cultures, Zobell Marine Broth 2216 (ZO) and Brain Heart Infusion Broth (BHI), and were maintained for 12 days at 28°C on a shaker (100 rpm). The ZO media was used for the growth of marine bacteria to target the marine microbial communities previously observed in coastal sinkholes (Suárez‐Moo et al., 2022) and BHI media was used as a generalist medium to culture different types of bacteria. When microbial growth was visible to the naked eye, 3 mL of microbial culture was collected for DNA extraction with a DNeasy PowerSoil Pro Kit (Qiagen). Negative controls for each treatment and culture medium used 50 mL liquid culture and 100 μL sterilized sinkhole water (Autoclaved for 15 min, 121°C, and 15 psi) as inoculum. No growth was observed in the controls; however, they were further tested for the presence of bacteria by a DNA extraction followed by 16S rRNA gene amplification. Only microbial cultures whose negative control lacked growth, with negative values of genomic DNA (measured in a NanoDrop™ One) and without PCR amplification (visualized in a 2% agarose gel), were used for the shotgun metagenomic sequencing.

In a previous metagenomic characterization of environmental samples (sediments) from Polac, we detected a high microbial diversity resulting in a low number of long contigs during metagenomic assembly, and therefore impairing BGC discovery. To reduce this diversity, we decided to employ enrichment cultivation methods to assemble longer contigs with that allowed better BGC characterization. In total, six DNA samples from the cultivation‐dependent approach (ORBHI, P1BHI, P2BHI, ORZO, P1ZO, P2ZO) and one environmental sample (PolAc54M) were subjected to quality control (Qubit Fluorometer), library preparation, and shotgun sequencing by Novogene, on a NovaSeq. 6000 platform (Supporting Information: Table A1).

### 2.2 Bioinformatic analysis of the MGs and MAGs

The quality of the reads was checked with FastQC version 0.11.2 (Andrews, 2010) and multiQC (Ewels et al., 2016), and low‐quality bases (per base sequence quality <33) were removed with Trimmomatic version 0.32 (Bolger et al., 2014). All sequencing files with high‐quality reads from the six cultures and the environmental sample were assembled with Megahit version 1.1.2 (Li et al., 2017). After contigs assembly, only contigs from metagenomes (MGs) with lengths >2000 bp were kept to avoid ambiguous gene annotation and binning errors from shorter contigs using BBMap version 37.93 (https://sourceforge.net/projects/bbmap/). Bwa version 0.7.15 (Li & Durbin, 2009) was used to map reads back to the assembled contigs, and SAMtools v.1.9 (Li et al., 2009) was used to convert files to binary format for further downstream analyses. MAGs were constructed with MetaBAT2 version 2.12.1 (Kang et al., 2019), and the quality of binned genomes was determined with CheckM version 1.1.3 (Parks et al., 2015). The completeness and contamination of the MAGs were checked against the standard established by the Genomic Standards Consortium (Bowers et al., 2017) and the high‐quality MAGs (>90% completeness and <5% contamination) were analyzed. High‐quality MAGs and the MGs were then assigned taxonomic identities (to genus level) with GTDB‐TK v2 (Chaumeil et al., 2020) and Kraken 2 (Wood & Salzberg, 2014), respectively. The abundance of the genera (sum of contigs by genus) from the MGs was computed with Braken2 (Lu et al., 2017) and default parameters against the GTDB 207 release were used in the GTDBTK analysis. High‐quality MAGs and MGs were used for the BGC and SM identification with antiSMASH 6 (Blin et al., 2021), NaPDoS (Ziemert et al., 2012), and Bagel4 (van Heel et al., 2018). (SMs refer to BGCs which matched identified SMs in the databases.) The antiSMASH pipeline can make predictions for a diverse range of SM pathways and all major natural product classes (Blin et al., 2019). The NaPDos pipeline compares sequences of PKS‐derived ketosynthase (KS) and NRPS‐derived condensation domains to a database of previously characterized natural products from NRPS and PKS gene clusters (Ziemert et al., 2012). BAGEL4 identifies and visualizes the Ribosomally synthesized and posttranslationally modified peptides (RiPPs) and bacteriocin BGCs, based on a core peptide database and hidden Markov models (HMMs) (van Heel et al., 2018).

A phylogenomic tree of high‐quality MAGs was generated with GToTree (Lee, 2019). A bacteria‐specific single‐copy gene (SCG) set that is available within the GToTree package was used with a phylogenetically broad set of genomes downloaded from NCBI (Supporting Information: Table A2). The phylogenomic tree was visualized with iTOL (Letunic & Bork, 2019).

Taxonomic diversity at the genus level based on contig abundance from MGs and the number of MAGs by cultivation treatment was visualized with plots using R Studio (Team, 2015). To visualize shared and exclusive genera between metagenomes Upsetplot was constructed by use of R package UpsetR (Lex et al., 2014). To examine the data distribution of the BGCs length in MGs and MAGs, histograms were constructed with the R Package “moments” in Rstudio (Team, 2015). Boxplots of the number of BGCs found in the 35 MAGs by antiSMASH, NaPDoS, and Bagel4 were generated with the R package ggplot2 (Ginestet, 2011). The abundance of the BGCs found in MAGs was used to generate heatmaps using the function heatmap.2 from the R package gplots v.3.0.1 (Warnes et al., 2016). The taxonomic diversity associated with each BGC type found in the high‐quality MAGs and assigned with GTDB‐TK (Chaumeil et al., 2020) was visualized with a barplot in Rstudio (Team, 2015).

Due to the low detection of BGCs in the environmental sample and to further explore the biosynthetic potential of the microbial community in the Polac sediments, a mapping of the reads from the original environmental library (PolAc54M) against the different BGC contigs (called BGC‐contig) from the culture‐based approach (metagenomes and high‐quality MAGs) was performed using the software bwa (Li & Durbin, 2009). To get an estimate of the mean coverage of each type of BGC‐contig, the following formula was used: (Depth of coverage) × (Number of bases on BGC‐contig)/(Length of the BGC‐contig). A heatmap from the R package gplots (Warnes et al., 2016) was used to visualize the mean coverage for each type of BGC.

A comparison between the diversity in microbial sediment communities from coastal sinkholes was performed between the PolAc54M (present study) and Zapote (Suárez‐Moo et al., 2022) samples. A subset of 20 million reads of the metagenomic shotgun data (PolAc54M) was extracted using vsearch v2.14.1 (Rognes et al., 2016) and was then aligned against a nonredundant version of the SILVA database v138 with an E‐value < 10−5. Sequences that matched this database were aligned against archaeal and bacterial 16S rRNA HMMs using ssu‐align 0.1.1 (https://github.com/EddyRivasLab/ssu-align). The 16S rRNA gene metabarcoding data used for “Zapote” belonged to a previous study (Suárez‐Moo et al., 2022) and were taxonomically assigned against the SILVA taxonomy database. Shared and unique taxa (from phylum to genus) were visualized with Venn diagrams using the R package VennDiagram v.1.6.20 (Chen & Boutros, 2011). Barplots were constructed to visualize the relative abundance of the 20 most abundant taxa in each sinkhole using Rstudio (Team, 2015).

## 3 RESULTS

### 3.1 Taxonomic and functional analysis of the metagenomes (MGs)

**Figure 2:**
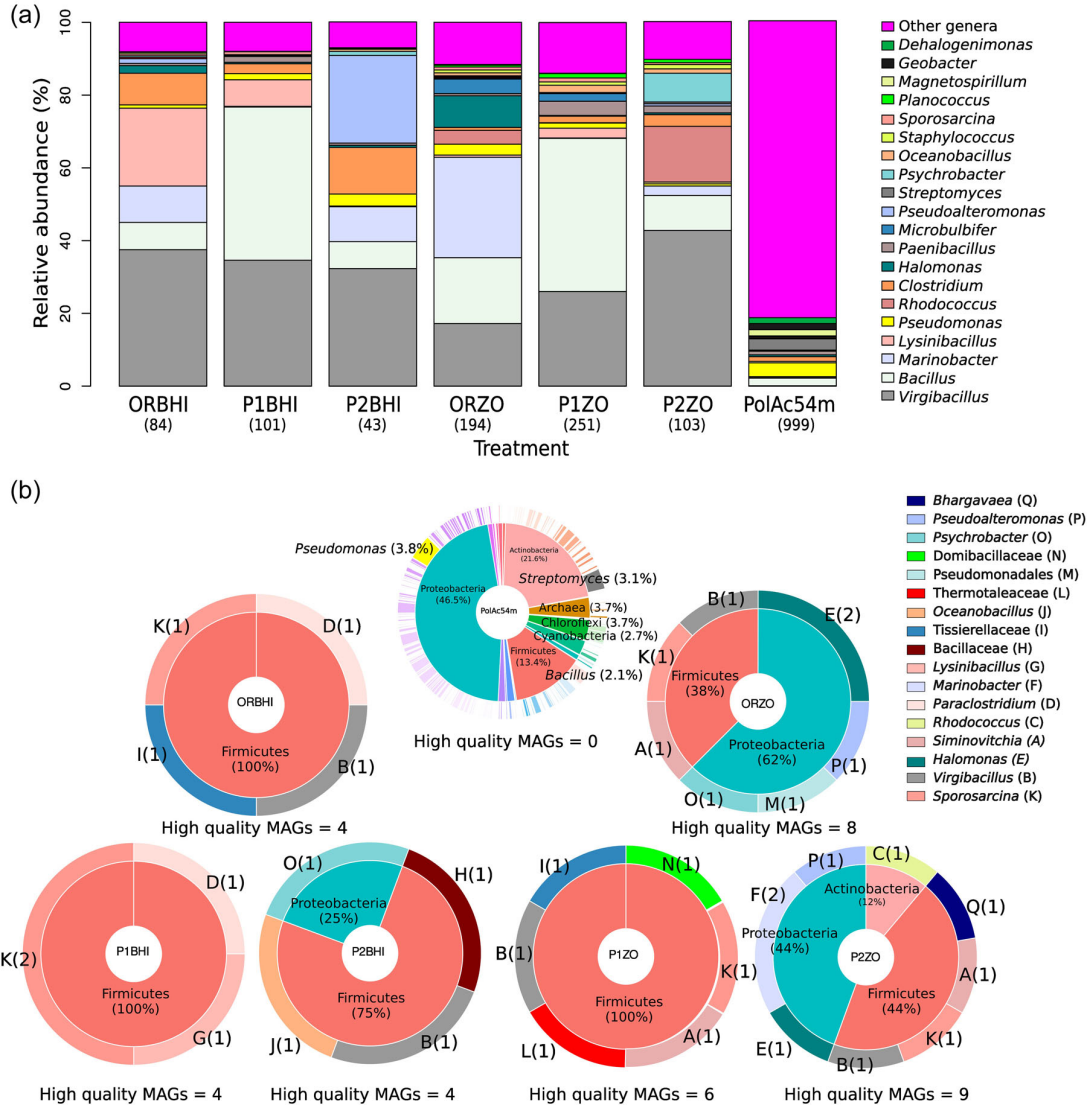
Taxonomic diversity of MGs and MAGs. (a) The 20 most abundant genera in the MGs. (b) Taxonomic diversity found in the MAGs. The number under the sample name and in parenthesis represents the genera found in each MG. Or, Original sediment culture; P1, pretreatment 1; P2, pretreatment 2; Zo, Zobell medium. The number in parenthesis in the PieDonut represents the number of MAGs associated with the taxa (letters and colors) and the percentage of contigs associated with the genus in the PolAc54M sample.

In total, 170.4 M reads were obtained from seven shotgun metagenomic libraries of the sediment sample from the coastal sinkhole (Figure 1, Supporting Information: Table A1). After quality filtering with trimmomatic, 161.5 M reads with a good Phred score (*Q* > 33, Supporting Information: Figure A1) were analyzed, with a range from 20.8 to 32.5 M reads per metagenome and an average of 23.1 M reads (Supporting Information: Table A1). Thirty‐eight phyla, 77 classes, 169 orders, 359 families, and 1023 genera were found in the seven metagenomes. For all metagenome, the most abundant phyla (percentage of the total reads in the seven libraries) were Firmicutes (56.9%), Proteobacteria (28.8%), Actinobacteria (6.6%), Bacteroidetes (1.6%), Euryarchaeota (1%), and Chlorofexi (1%). The most abundant classes were Bacilli (53%), Gammaproteobacteria (19%), and Actinobacteria (6.6%). Of 169 orders, the most abundant were Bacillales (50.3%), Alteromonadales (7.4%), and Clostridiales (4.8%). The Three most abundant families were Bacillaceae (46.2%), Alteromonadaceae (6.1%), and Clostridiaceae (2.3%) (Supporting Information: Figure A2). At the genus level, there was no predominance associated with the treatment or type of culture medium. However, the number of genera was lower in the cultivation‐dependent approach (Supporting Information: Figure A3) and some genera were dominant, namely *Virgibacillus* with (percentage of the total reads by metagenome) a range of 17.2% in ORZO to 42.8% P2ZO, and *Bacillus* with a range of 7.4% in P2ZO to 42.1% in P1BHI (Figure 2a). The environmental sample was the most genera‐rich sample, with 999 genera, the most abundant being *Pseudomonas* (6.4%), *Streptomyces* (5.3%), and *Bacillus* (3.6%) (Figure 2a). This sample had the highest number of unique genera with 704 followed by P1ZO with 6 and P1BHI with 3 (Supporting Information: Figure A3).

The search for the biosynthetic potential of the metagenomes detected 3 BGCs within 1 BGC type (arylpolyene) in the environment sample compared with 489 BGCs from within 32 BGC types in the 6 cultivation‐dependent metagenomes using antiSMASH, which ranged from 56 in P2BHI to 135 in P1ZO (Supporting Information: Figure A4 and Supporting Information: Table A3). Overall, no differences could be attributed to pretreatment of the sediments or culturing medium (Supporting Information: Figure A4). Nonribosomal peptide‐synthetase (NRPS), Type III polyketide synthase (T3PKS), and terpene were the most abundant BGC types for cultivation‐dependent metagenomes (Figure A4). P1ZO showed the highest number of BGCs with 135 from within 24 BGC types (Supporting Information: Table A3). Despite the low detection of BGC in the environment sample, some reads of this library were mapped against the BGCs‐contig found in the metagenomes and MAGs. BGCs such as T1PKS and LAP mapped against the PolAc54Mmetagenome had the highest values of the coverage mean with 0.99 and 0.73, respectively, while ectoine mapped against the high‐quality MAGs had a coverage mean of 0.61 in ORZO (Supporting Information: Figure A5).

The diversity comparison between sediment microbial communities found in two coastal sinkholes (Supporting Information: Figure A6) revealed that both sinkholes shared taxa 13% of their genera, corresponding to 67.7% of their phyla (Supporting Information: Figure A6). The sediment microbial community structure varied between the coastal sinkholes and it only showed similar relative abundances in a few of the most abundant taxa such as *Desulfatiglans* (Supporting Information: Figure A6).

### 3.2 Taxonomic diversity in the high‐quality MAGs

To further investigate this potential source of novel SMs, we sought to reconstruct novel genomes from these metagenomes. Of the 82 MAGs obtained after the binning process, 35 MAGs were considered high‐quality according to established standards (≥90% completeness, ≤5% contamination) (Supporting Information: Tables A1 and A4). The sole MAG obtained from the PolAc54M metagenome was not used for any downstream analyses since it failed to meet good quality standards (low N50 value, 0 completeness, 99% contamination). These results justified the pretreatment of the sediments and exposure to high‐nutrient culture media to reduce the microbial diversity and explore the biosynthetic potential of a higher number of complete genomes. The P2ZO metagenome had the greatest recovery of high‐quality MAGs with 9, while the ORBHI, P1BHI, and P2BHI treatments recovered 4 MAGs each (Supporting Information: Table A1).

For the 35 high‐quality MAGs recovered from the cultivation‐dependent metagenomes, 29 were assigned to a genus level (12 bacterial genera), 5 to a family level (4 bacterial families), and 1 to an order level (1 bacterial order) (Figure 2b, and Supporting Information: Table A4). Most of the MAGs belonged to the phylum Firmicutes, followed by Proteobacteria and Actinobacteria (Figure 2b). The most abundant genera recovered as MAGs from all treatments were *Sporosarcina* (6 MAGs), *Virgibacillus* (5 MAGs), *Halomonas* (3 MAGs), and *Siminovitchia* (3 MAGs) (Figure 2b). *Rhodococcus* was the only genus belonging to the phylum Actinobacteria and was found in a MAG from the P2ZO metagenome (Figure 2b).

### 3.3 Functional diversity in the MAGs

**Figure 3:**
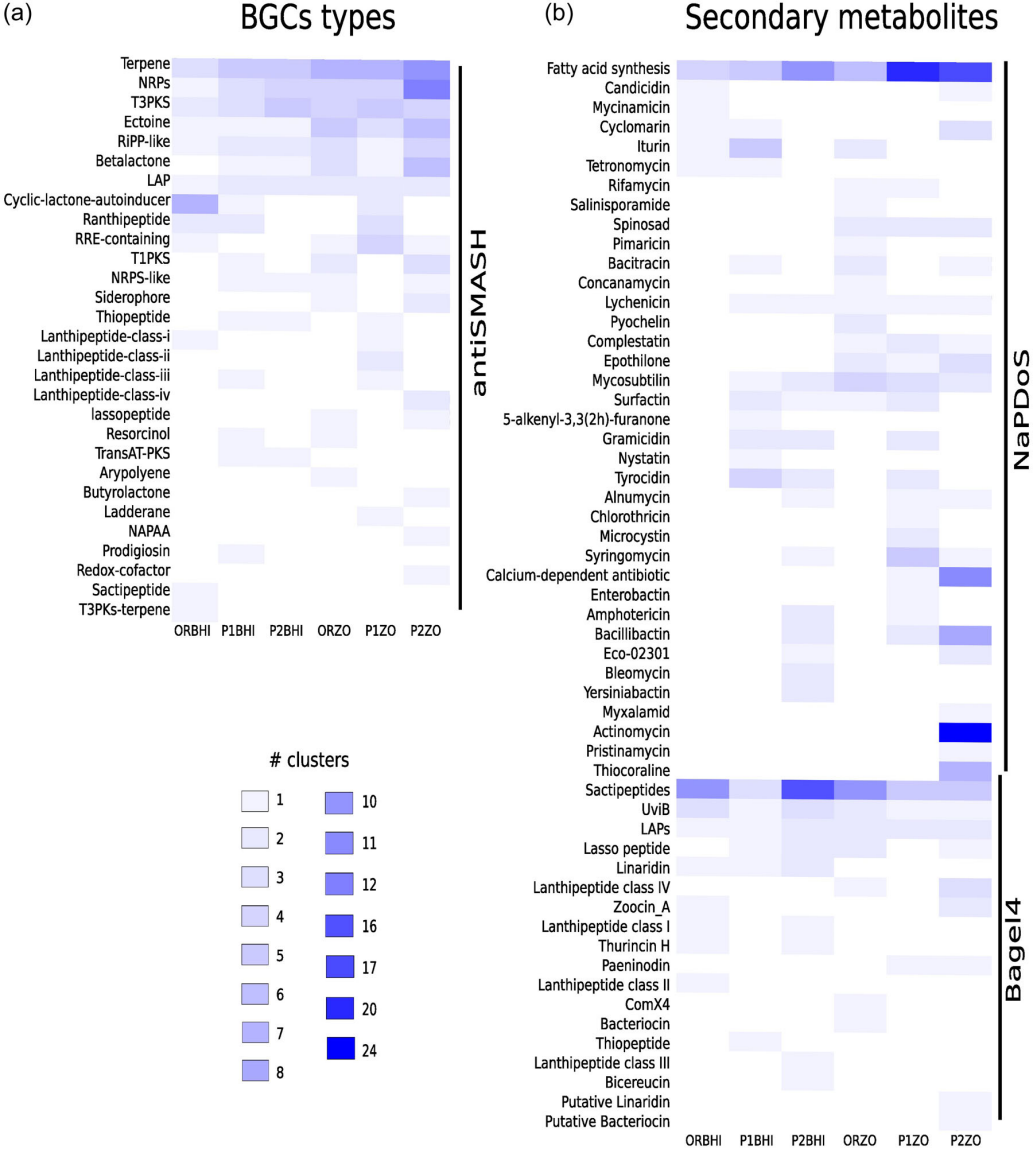
Detection in the 35 high‐quality MAGs of (a) BGCs by antiSMASH, and (b) SMs by NaPDoS, and Bagel4. Gene clusters are arranged top to bottom by the abundance of BGC‐type.

The 35 high‐quality MAGs were analyzed with antiSMASH, NaPDoS, and Bagel4 to discover their biosynthetic potential (Figure 3). The abundance of BGCs and SMs found in all MAGs belonging to each metagenome was visualized by metagenome (Figure 3). Analysis by antiSMASH detected 203 BGCs in the MAGs from cultivation‐dependent metagenomes, with a range of 22 in MAGs from ORBHI to 57 in MAGs from P2ZO (Figure 3a). In this pipeline 29 BGC types were found, of which terpene, NRPS, and T3PKS were the most abundant, while RiPP‐like and LAP were the least abundant. Fifty‐one BGCs (25% of total BGCs) showed similarity with known clusters from the MiBIG database; however, most of the suggested SMs had low percentage gene similarity with previous reports of these natural products (Supporting Information: Table A5). The identified natural products with the closest similarity were ectoine with 100%, followed by paeninodin (80%), turnebactin (61%), heterobactin A/heterobactin S2 (54%), and fengycin (46%) (Supporting Information: Figure A7).

NaPDoS detected 227 SMs belonging to 37 SM types (ranging from 9 in MAGs of ORBHI to 87 in MAGs of P2ZO) (Figure 3b). Fatty acid synthesis and actinomycin were the most abundant, while myxalamid and pristinamycin were the least abundant. Bagel4 detected 101 SMs of 18 SM types in high‐quality MAGs (ranging from 8 in MAGs of P2BHI to 29 in MAGs of P1ZO) (Figure 3b). Sactipeptides and UviB were the most abundant and Putative Linaridin and Putative Bacteriocin the least. The functional composition, like the genera, did not show any influence of treatment or culture medium on the relative abundance of BGC or SM types (Figure 3 and Supporting Information: Figure A8).

### 3.4 Phylogenomic analysis of the biosynthetic potential

**Figure 4:**
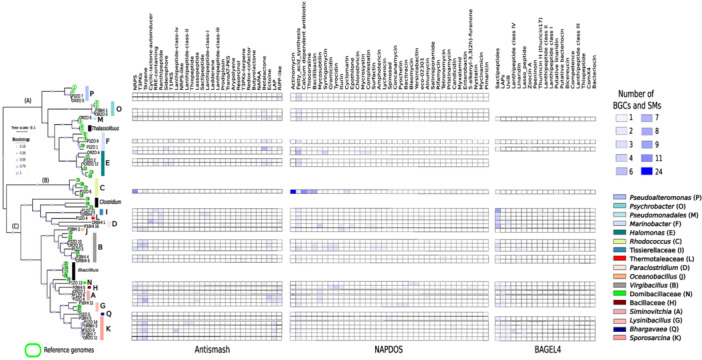
Phylogenetic and metabolic representation of assembled bacterial MAGs. Phylogenomic analyses for the MAGs and reference genomes used a bacteria‐specific single‐copy gene (SCG) set (86 markers) in the GToTree program. MAGs were visualized with functional annotation by AntiSMASH, NaPDoS, and Bagel4. Green circle; reference genome. Number within the circle: number of the respective genome (see Supporting Information: Table A2). Letters (A, B, C) indicate the phylogenetic clusters.

To test for possible phylogenetic patterns that would suggest the specificity of a BGC for a bacterial MAG, the MAGs and reference genomes were placed in a phylogeny constructed using a Bacteria‐specific SCG set to the genus level with the BGCs and SMs found with the antiSMASH, NaPDoS, and Bagel4 pipelines (Figure 4). The phylogenetic GToTree analysis shows three clades: (A) included 10 MAGs, of which two were classified as *Pseudoalteromonas*, two as *Psychrobacter*, one as Pseudomonadales, two as *Marinobacter*, and three as *Halomonas*; (B) included only one MAG, classified as *Rhodococcus*; and (C) included a subclade with two MAGs classified as Tissierellaceae, one as Thermotaleaceae, and two as *Paraclostridium*, and another subclade with one as *Oceanobacillus*, five MAGs classified as *Virgibacillus*, one as Domibacillaceae, one as Bacillaceae, three as *Siminovitchia*, one as *Lysinibacillus*, one as *Bhargavaeae*, and six MAGs classified as *Sporosarcina*.

Clade C had the highest abundance and diversity of BGCs and SMs identified by antiSMASH (146 BGCs in 24 types), NaPDoS (112 SMs in 30 types), and Bagel4 (94 SMs in 17 types) (Figure 4). It also had a high number of unique BGC and SM types identified by antiSMASH (15 BGCs), NaPDoS (17 SMs), and Bagel4 (15SMs), of which T3PKS and LAP were the most abundant BGCs, while iturin and tyrocidin were the most abundant unique SMs identified by NaPDoS. UviB and LAPs were the most abundant unique SMs with Bagel4. Clade B had three unique BGCs (butyrolactone, NAPAA, and redox‐cofactor), three unique SMs (actinomycin, thiocoraline, and pristinamycin) identified by NaPDoS, and two unique SMs (putative Mvan 2782 linaridin, and putative bacteriocin‐family protein) by Bagel4 (Figure 4).

Differences in abundance and diversity of BGCs and SMs were observed among very closely related strains (Figure 4). The MAGs P1ZO_21 and ORBHI_7 belonging to the family Tissierelaceae shared two BGC types, one SM (by NaPDoS), and two SMs (by Bagel4); however, each MAG had two BGC and one SM (NaPDoS) that were unique. The MAGs P2ZO_10 and ORZO_10 belonging to the genus *Virgibacillus* did not share any SM type, while ORZO_10 had nine unique SM types in the NaPDoS analysis (Figure 4).

### 3.5 Taxonomic analysis of the biosynthetic potential

**Figure 5:**
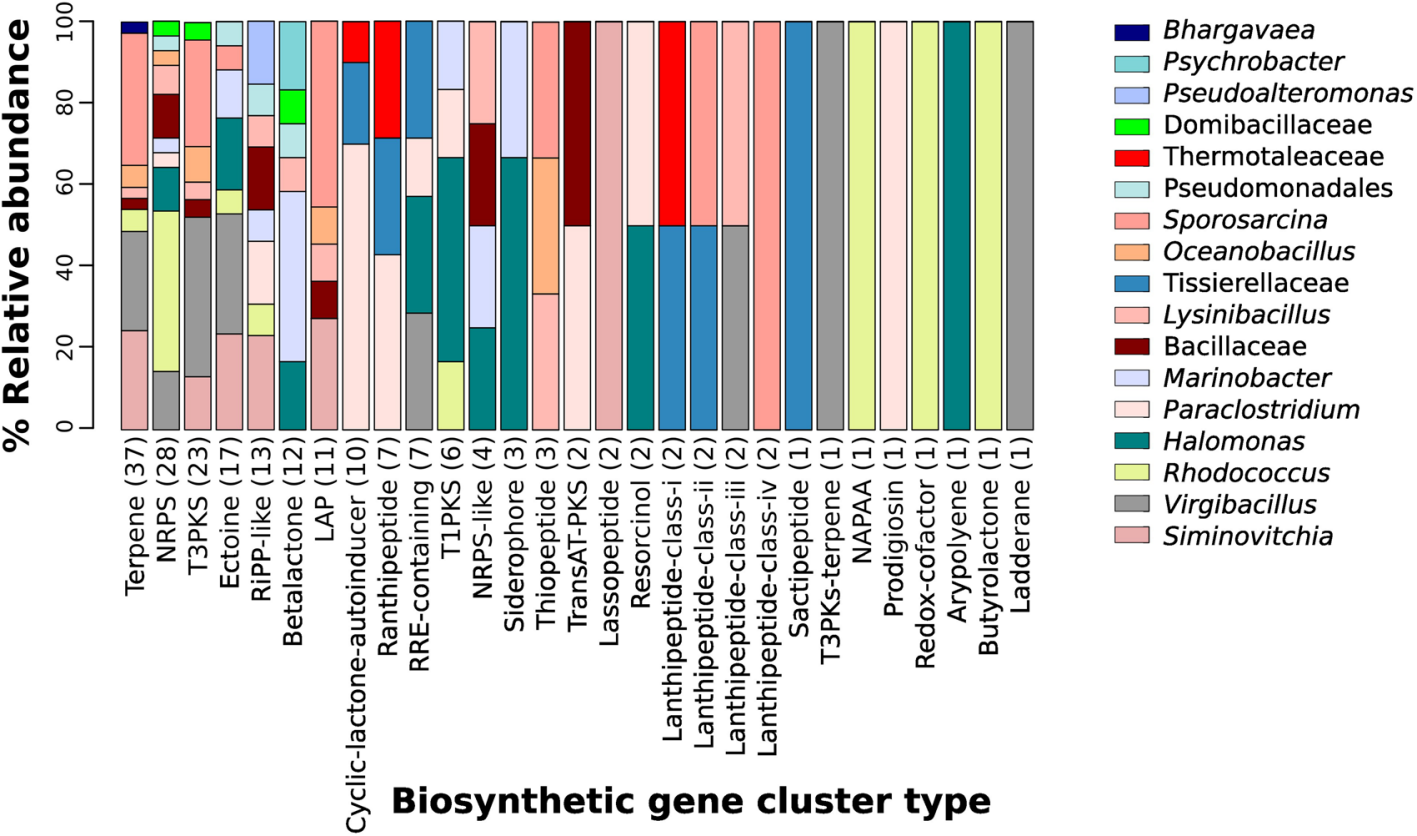
Assigned taxonomic diversity of the BGCs detected in 35 high‐quality MAGs by antiSMASH. Parentheses represent the number of BGCs found in the 35 MAGs.

We found 203 BGCs representing 29 types of compounds in the 35 high‐quality MAGs (Figure 5). The genus with the most number of BGCs assigned was *Virgibacillus* with 32 (16%), followed by *Sporosarcina* with 28 (14%), *Siminovitchia* with 24 BGCs (12%), *Rhodococcus* with 19 (9%), *Halomonas* with 18 (9%), and *Paraclostridium* with 18 (9%). The genera *Halomonas, Paraclostridium*, and *Lysinibacillus* had the most types of BGCs assigned with nine BGC types each (Figure 5). *Virgibacillus* had the most T3PKS, ectoine, Lanthipeptide‐class‐iii, T3PKs‐terpene, and ladderane BGCs assigned. *Sporosarcina* had the most Terpene, and LAP BGCs assigned. *Siminovitchia* had the RiPP‐like and lassopeptide BGCs assigned. *Rhodococcus* had the most NRPS, NAPAA, Redox‐cofactor, and butyrolactone BGCs assigned (Figure 5).

## 4 DISCUSSION

This study focused on the recovery of MAGs and BGCs from sediment microbial communities from a sinkhole in the Yucatan peninsula. Previous studies in this ecosystem used 16S rRNA amplicons (Brankovits et al., 2017; Moore et al., 2020; Suárez‐Moo et al., 2022) and shotgun metagenomics of environment samples (Marfil‐Santana et al., 2016; Moore et al., 2020) to explore mainly the taxonomic and functional diversity of the microbial communities from the Yucatan. A comparison between the diversity in sediment microbial communities of environmental samples from coastal sinkholes (PolAc54M vs. El Zapote) revealed a low number of shared genera (13%). Sediment microbial community structure also varied between sinkholes and only the sulfate‐reducing bacteria (SRB) belonging to the genus *Desulfatiglans* (Suzuki et al., 2014) was abundant in both sinkholes. The 16S rRNA genes recovered from the PolAc54M metagenome and the taxonomic annotation of metagenomic reads suggest there is a high abundance of families associated with sulfate reduction (Desulfobulbaceae, Desulfobacteraceae, Desulfatiglandaceae, Desulfosarcinaceae, Desulfobaccaceae, Desulfococcaceae) (Suárez‐Moo et al., 2022; Zhang et al., 2022), and genera associated with ammonia oxidation (*Nitrospira*) (Jiang et al., 2023) and denitrification (*Pseudomonas* and *Bacillus*) (Arat et al., 2015; Yang et al., 2020). Though the investigation of microbial communities and their role in biogeochemical cycles were not the goals of this study, it is interesting to highlight that the presence of these microorganisms in sinkhole environments suggests they hold important roles in sulfate reduction, ammonia oxidation, and denitrification (Suárez‐Moo et al., 2022). Further research on the differences and roles of these communities in biogeochemical cycles should involve a higher number of sinkholes in diverse locations of the Yucatan peninsula.

More importantly, results from the present study suggest that the implementation of liquid cultures using an *inoculum* of environment sample along with a previous treatment (drying and temperature) leads to a reduction of diversity, which in turn allows for a higher resolution of the genomes present after the selective treatment is applied, leading to a better understanding of the SM pathways they possess. Cultivation‐dependent systems could promote ecological interactions between the culture members such as antagonism, cooperation, and synergism that could activate the biosynthesis of natural products that are not easy to induce otherwise (Li & Rebuffat, 2019; Zhuang & Zhang, 2021). A cultivation‐based approach leads to a sharing of growth factors with neighboring species, allowing the cultivation of microorganisms unable to grow in isolation (D'Onofrio et al., 2010). On the other hand, the high microbial diversity found in the environmental metagenome and the limitations of short reads by Illumina (Chen, Chiang, et al., 2021) do not yield the long contigs necessary for good genome mining, and a low biosynthetic potential was detected in this microbial community. BGC identification in environmental samples requires an efficient method of BGC identification such as hybrid‐assembled contigs (long‐ and short‐read sequencing) (Chen, Chiang, et al., 2021; Negri et al., 2022).

### 4.1 Sediment from the Polac sinkhole has a high abundance and diversity of BGCs and SMs

Terpene, NRPS, and PKS gene clusters were the most abundant in the MAGs of the present study. Similar results have been observed in previous studies of MAGs from several environments such as microbial mat layers (Chen, Wong, et al., 2020), outdoor pier surfaces (Tong et al., 2021), and the water of lake environments (Chen, Chiang, et al., 2021; Cuadrat et al., 2018). Terpenes, as well as NRPs and PKS, were the most abundant clusters in diverse habitats representing all continents and oceans and with a wide distribution in diverse microbial phyla (Nayfach et al., 2021). These three BGC types have been recorded as the most common within marine bacteria (Moghaddam et al., 2021). A simple explanation is that the genes associated with these BGCs, such as those coding terpene synthases, are widely distributed in bacterial genomes (Yamada et al., 2015). However, there may be a bias in the status of the antiSMASH database, with NRPS (23%), PKS (17%), and terpene synthases (12%) being the most abundant BGCs from the total of 172,395 BGCs from 24,776 unique species/strains present in this pipeline (Moghaddam et al., 2021). A high BGC and SM diversity was found in the Polac sinkhole sediment, higher than the 10 BGC types reported for mangrove sediments that lie very close to Polac and that were studied using cultivation‐dependent metagenomes (Marfil‐Santana et al., 2021); of those BGC types, eight were found in our metagenomes, including terpenes and NRPS. That study reported 23 SMs of which 8 were detected in Polac's MAGs.

The high diversity of BGCs and SMs found in this subcommunity from sinkhole sediments might be partly attributable to the ecological function of the natural products they produce which may enable the observed microbial community to locally adapt to the unique conditions generally found in the karst sinkholes from the Yucatan (e.g., high concentrations of organic and inorganic carbon, high concentrations of total nitrogen, and saline intrusion from the ocean) (Suárez‐Moo et al., 2022). The high diversity of BGCs and SMs found in this cultivation experiment suggests that microbes from this enriched environment may be using these molecules to outcompete the highly abundant Proteobacteria (46.5%) and Actinobacteria (21.6%) found in the original microbial community. Moreover, the reads from the environmental sample library could be mapped against the BGCs contigs found in the cultivation‐dependent metagenomes and high‐quality MAGs, some of them with high coverage mean values, suggesting that the biosynthetic potential found in the microbial community recovered by the cultivation experiment was representative of the natural microbial community and that cultivation experiments are a useful way to enhance the recovery of the biosynthetic potential originally observed in environmental metagenomes.

Regarding the kind of BGCs recovered in the MAGs, terpenes, ectoine, and siderophores have been described as molecules with biological role(s) for the adaptation of the microbial communities against harsh environmental conditions (Abdel‐Mageed et al., 2020; Chen, Wong, et al., 2020; Sayed et al., 2020). BGCs associated with ectoine were found in high abundance in most of the six cultivation‐dependent metagenomes and the MAGs assembled from them. Contigs of this BGC found in the high‐quality MAGs were mapped against the environmental sample, and coverage mean values had an average of 0.11, with the MAGs from the ORZO experimental metagenome showing the highest value (c. 0.61). These BGCs have been associated with osmoprotectant metabolites which serve to protect cells from osmotic stress in high‐salinity environments (Czech et al., 2018; Reshetnikov et al., 2011). We hypothesized that the high salinity present in the cave of the Polac sinkhole has led to an adaptation in members of the Polac microbial community to encode ectoine gene clusters.

Siderophores (pyochelin, enterobactin, bacillibactin, yersiniabactin) were found in the MAGs and their presence has also been reported in bacteria strains isolated from water and sediment from sinkholes in the peninsula (De La Rosa‐García et al., 2007) These low molecular weight compounds have a high binding affinity for insoluble Fe(III) (D'Onofrio et al., 2010)⁠ and allow the survival and proliferation of microorganisms in free iron‐deficient environments (Wang & Li, 2022). Iron concentrations in the sediment of Polac sinkhole were not measured; however, very low concentrations of dissolved iron (0.1 and 0.01 µmol L^−1^) were reported in fresh water from another coastal sinkhole on this peninsula (Ritter et al., 2019), which supports the idea that the siderophores could be important for the survival of the microbial community from this environment.

Our study revealed that most of the identified BGCs (75%) showed no sequence homology with bacterial BGCs reported previously in the antiSMASH‐MiBiG pipeline, and only a few BGCs showed even a low level of similarity with their reported SMs; this suggests that the novel BGCs found in our study (*n* = 152) could encode new specialized compounds. Similar approaches to BGC exploration have yielded novel BGCs in microbial communities and isolated strains from environments such as microbial mat layers (Chen, Wong, et al., 2020), the water of a meromictic lake (Chen, Chiang, et al., 2021), and water of the Arctic Ocean (Rego et al., 2021).

New approaches to BGC identification will be required because some bacteria can produce far greater numbers of SMs than can be revealed by conventional screening (Sekurova et al., 2019) or that can be seen under laboratory conditions (Scherlach & Hertweck, 2021). This study supports the use of contemporary genomic and bioinformatic tools together with isolation methods to increase the diversity of BGCs and SMs that can be studied in a microbial community from new environments (Scherlach & Hertweck, 2021; Sekurova et al., 2019). We developed our study to detect BGCs and SMs in surface sediment subcommunities grown in liquid cultures under high nutrient conditions; therefore, evaluation of the effects that these molecules have in the natural microbial communities will need consideration of the local nutrient and environmental conditions (Pishchany & Kolter, 2020). Further research associated with the BGCs discovery in karst ecosystems by genome mining should be accompanied by long‐read sequencing to obtain unfragmented genomic assemblies that lead to better identification of complete BGCs.

### 4.2 Taxonomy of the biosynthetic potential found in MAGs

Most of the genera associated with BGCs belonged to phylum Firmicutes, followed by Proteobacteria and Actinobacteria, the latter two are the most common contributors to marine bacterial natural products while Firmicutes are considered the fifth phylum in this rank (Williams, 2009). However, many biosynthetic genes for natural products (mainly PKS and NRPS) are widespread among members of the Firmicute (Letzel et al., 2013). The exploration of BGCs in microbial species that belong to other phyla different from Actinobacteria as Firmicutes could lead to an enhanced probability of success for the discovery of new bioactive natural products.

At the genus level, *Virgibacillus* showed the highest number of BGCs. These MAGs had BGCs mainly associated with T3PKS, ectoine, Lanthipeptide‐class‐iii, T3PKs‐terpene, and ladderane. T3PKS, ectoine, and lanthipeptide were also abundant in genomes of *Virgibacillus* species isolated from mangrove mud in the Red Sea (Othoum et al., 2019). Previous studies of genome mining and activity assays in *Virgibacillus* strains isolated a lanthipeptide named virgicin, which had inhibitory activity against the growth and biofilm formation of *Enterococcus faecalis* (Gupta et al., 2019). Galaviz‐Silva et al. Isolated *Virgibacillus* strains from marine ecosystems in Mexico and found antagonistic activity toward the food‐poisoning agents *Staphylococcus aureus* and *Vibrio parahaemolyticus*. (Galaviz‐Silva et al., 2018).

The genus *Siminovitchia* showed a high biosynthetic potential in our genome mining. This genus is a novel genus subjected to a reclassification from *Bacillus* based on strong phylogenetic and molecular evidence (Gupta et al., 2020), therefore, there is little in silico and experimental information about the SMs it produces. In our results, we found that this genus dominated several BGC types such as RiPP‐like and lassopeptide, and has the potential to produce SMs, such as mycosubtilin, alnumycin, and paeninodin. Our results suggest that, just like *Bacillus*, the genus *Siminovitchia* can produce SMs with surfactant and antimicrobial activities (Kaspar et al., 2019; Khatoon et al., 2022). Within the Actinobacteria, our enrichment methods only recovered MAGs from the genus *Rhodococcus*; which had a great number of NRPSs, as do previously described genomes of *Rhodococcus* strains isolated from several environments where NRPS, PKs, terpenes, and butyolactone have been reported as the dominant types of SMs (Ceniceros et al., 2017; Doroghazi & Metcalf, 2013). In these studies, differences in the number and diversity of secondary metabolite BGCs were associated with the type of environment (soils vs. marine and animal host) (Doroghazi & Metcalf, 2013) and phylogenetic clade of the strains (Ceniceros et al., 2017). Bacterial strains belonging to this genus have been characterized as sources of new natural products with antifungal, antibacterial, and antitrypanosomal activities (Elsayed et al., 2017).

High diversity and abundance of BGCs were found in the genus *Halomonas* with the antiSMASH analysis, including a great number of BGCs associated with ectoine. Different *Halomonas* species can resist environmental osmotic stress by synthesizing ectoine as a compatible solute, with an increase of the intracellular ectoine concentration with high NaCl concentration in the growth medium (Pastor et al., 2010). Strains belonging to *Halomonas elongata* have been used in the production of ectoine for cosmetic and medical industries (Liu et al., 2021). The genus *Sporosarcina* had the second highest abundance of BGCs, however, to date, there is no information about the BGCs diversity of this genus.

### 4.3 Phylogenetic distribution patterns of BGCs and SMs found in MAGs

Phylogenetically close MAGs belonging to the family Tissierellaceae and the genus *Virgibacillus* had unique BGCs, suggesting that closely related strains vary in their BGC composition, which may be indicative of recent gene loss and/or horizontal gene transfer events (Letzel et al., 2017; Steinke et al., 2021). This gene transfer provides a mechanism to extend BGC diversity (Chase et al., 2021). A study of BGC analysis of strains belonging to three species of the genus *Salinispora* from different locations reported that most of the BGCs (PKS and NRP) occurred in only one or two strains (Ziemert et al., 2014). Based on sequence identity (SI), the authors suggested that most of these BGCS had been acquired from other high‐G + C bacteria such as *Streptomyces* spp., resulting in enhanced adaptive capabilities for strains at each particular environment. This phylogenetic pattern has also been observed in the *Bacillus subtilis* group (Steinke et al., 2021), where the gene cluster families were specific to a species or clade.

We are aware of the limitations in the phylogenetic pattern analyses due to the conditions used during the assembly of the MAGs since the four compared MAGs had 95% completeness and <2.2 contamination. Short‐read‐derived MAGs usually comprise numerous short contigs in which genomic characters are missed (Chen, Chiang, et al., 2021). Further research on the influence of the phylogeny in the diversity and abundance of BGCs and SMs in the microbial communities of sediments in karst sinkholes should focus on the use of genomes from cultivated axenic isolates which lead to complete and noncontaminate genomes.

### 4.4 Biotechnological and medical importance of the BGCs and SMs found in the MAGs

Some natural products and BGCs found in silico in this search for BGCs have been associated with biological activities such as antibacterial, antifungal, and anticancer properties. Our results support early studies reporting the presence of bacterial strains isolated from water and sediments from sinkholes with inhibitory activity against bacteria and fungi pathogenic to humans and plants (De La Rosa‐García et al., 2007). Exploration in silico of microbial communities of water and sediments of the karst ecosystem on the Yucatán peninsula revealed multidrug resistance genes associated with antibiotics such as erythromycin, fosfomycin, streptothricin, indicating that these microbial communities may be producing these antimicrobials or are exposed to them in this environment (Moore et al., 2020).

Some SMs found in our MAGs such as Gramicidin, Pristinamycin, Rifamycin, Bacitracin, Thurincin H, and Bicereucin have been found to have antibacterial activity (Huo & Van Der Donk, 2016; Katz & Baltz, 2016; Ortiz‐Rodríguez et al., 2023; Singh et al., 2019; Sušnik & Koert, 2020). Other natural products BGCs found in our study such as Iturin, candicidin, Amphotericin, Nystatin, Pimaricin, thiocoraline, Syringomycin have been reported to have antifungal properties (Brautaset et al., 2008; Haeder et al., 2009; Katz & Baltz, 2016; Kawasaki et al., 2016; Qi et al., 2015; Son et al., 2016). Bleomycin, Actinomycin, Salinosporamide, and epothilone, also found in our mining efforts, found here, have been reported to show antitumoral activity (Erba et al., 1999; Katz & Baltz, 2016; Tang et al., 2000). Sactipeptides were the most abundant among the SMs bioprospected by the Bagel4 pipeline and have been associated with several beneficial properties for human health including clinical antimicrobial, anti‐inflammatory, spermicidal, and hemolytic properties (Chen, Wang, et al., 2021; Roblin et al., 2021).

The discovery of natural products with biotechnological and medical applications in the microbial communities from sediments in karst sinkholes is limited. The present work reveals a high diversity of BGCs and SMs that could lead to a bioprospecting strategy in this unexplored environment to identify and isolate new natural products with biological activity that may prove highly desirable in the present scenario of increasing antibiotic resistance among human pathogens.

## AUTHOR CONTRIBUTIONS

**Pablo Suarez‐Moo**: Conceptualization (equal); investigation (equal); formal analysis (lead); writing—original draft (equal); visualization (equal); writing—review and editing (equal). **Alejandra Prieto‐Davo**: Conceptualization (equal); investigation (equal); writing—original draft (equal); visualization (equal); writing—review and editing (equal); funding acquisition (lead); supervision (lead).

## CONFLICT OF INTEREST STATEMENT

None declared.

## ETHICS STATEMENT

None required.

## Supporting information

Supporting information.

Supporting information.

## Data Availability

All sequencing data are available in the NCBI repository under BioProject accession number PRJNA930412: https://www.ncbi.nlm.nih.gov/bioproject/PRJNA930412.
