# Supplementary material for: Biosynthetic potential of the sediment microbial subcommunities of an unexplored karst ecosystem and its ecological implications
Source: Microbiologyopen. 2024 Apr 9;13(2):e1407. doi: 10.1002/mbo3.1407 (PMC11003711; doi:10.1002/mbo3.1407)

**APPENDIX**

**
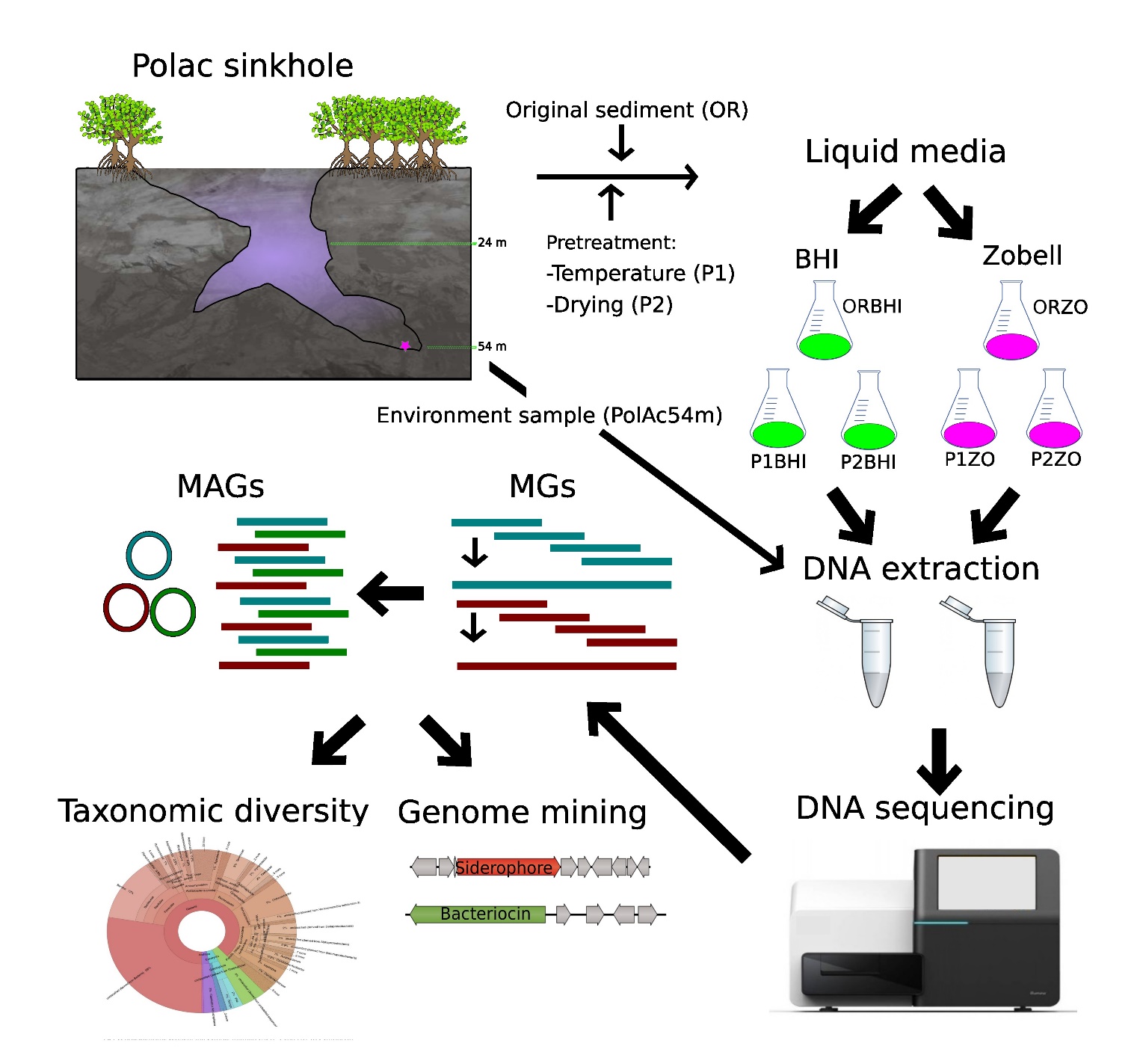
**

**Figure A0.** This study employed an enrichment method and genome mining tools to uncover novel bacterial genomes and explore their biosynthetic potential. The research focused on microbial communities residing in the sediments of an uncharted karst environment, specifically the Polac sinkhole, located on the Yucatán Peninsula.

**Fig. A1.** Phred score of the seven metagenomic shotgun libraries (green lines) after quality filtration by Trimmomatic.


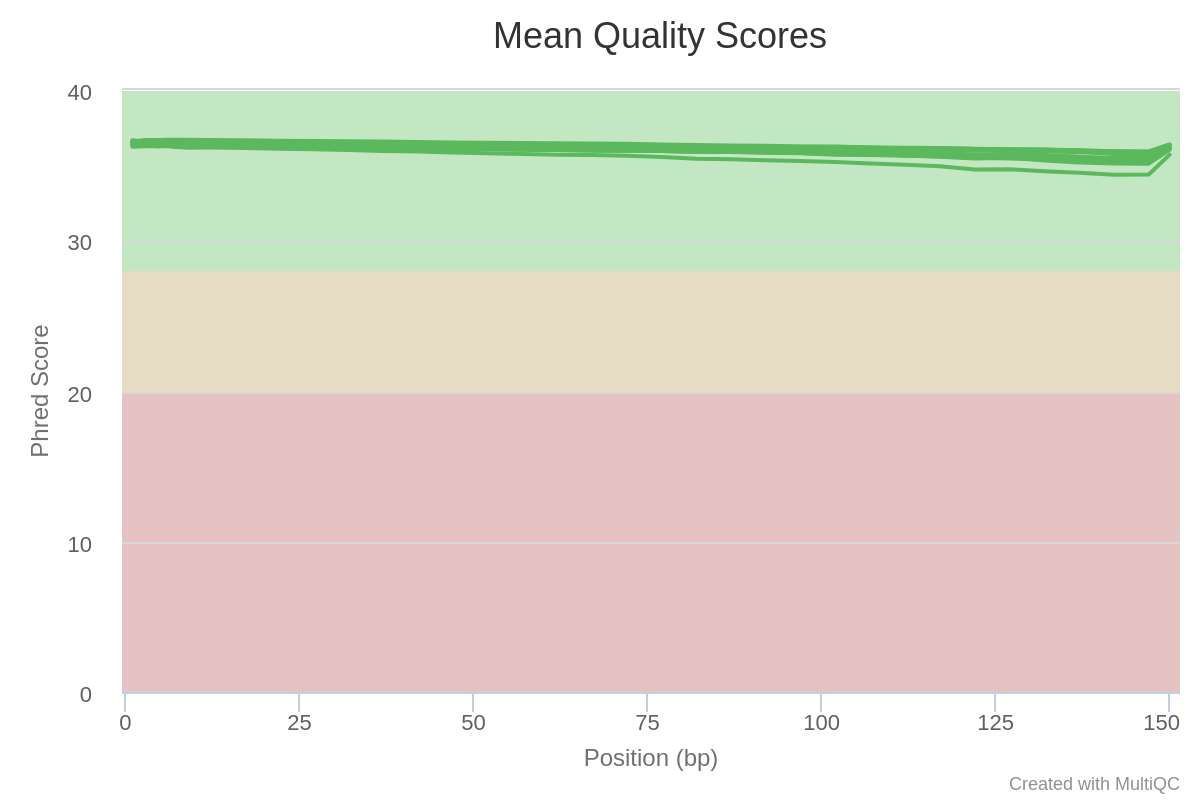


**Fig. A2.** Taxonomic diversity of the 20 most abundant taxa (from Phylum to family) found in the metagenomes. In parenthesis, the total found for each metagenome.


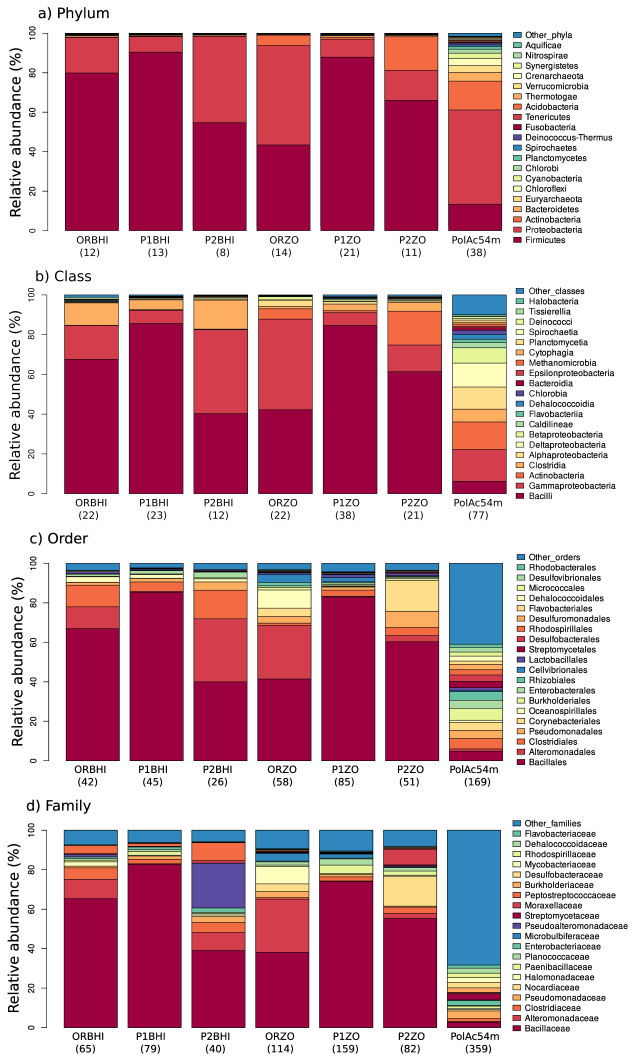


**Fig. A3.** Genera shared and unique between the environment sample (PolAc54m) and treatments in two liquid media.


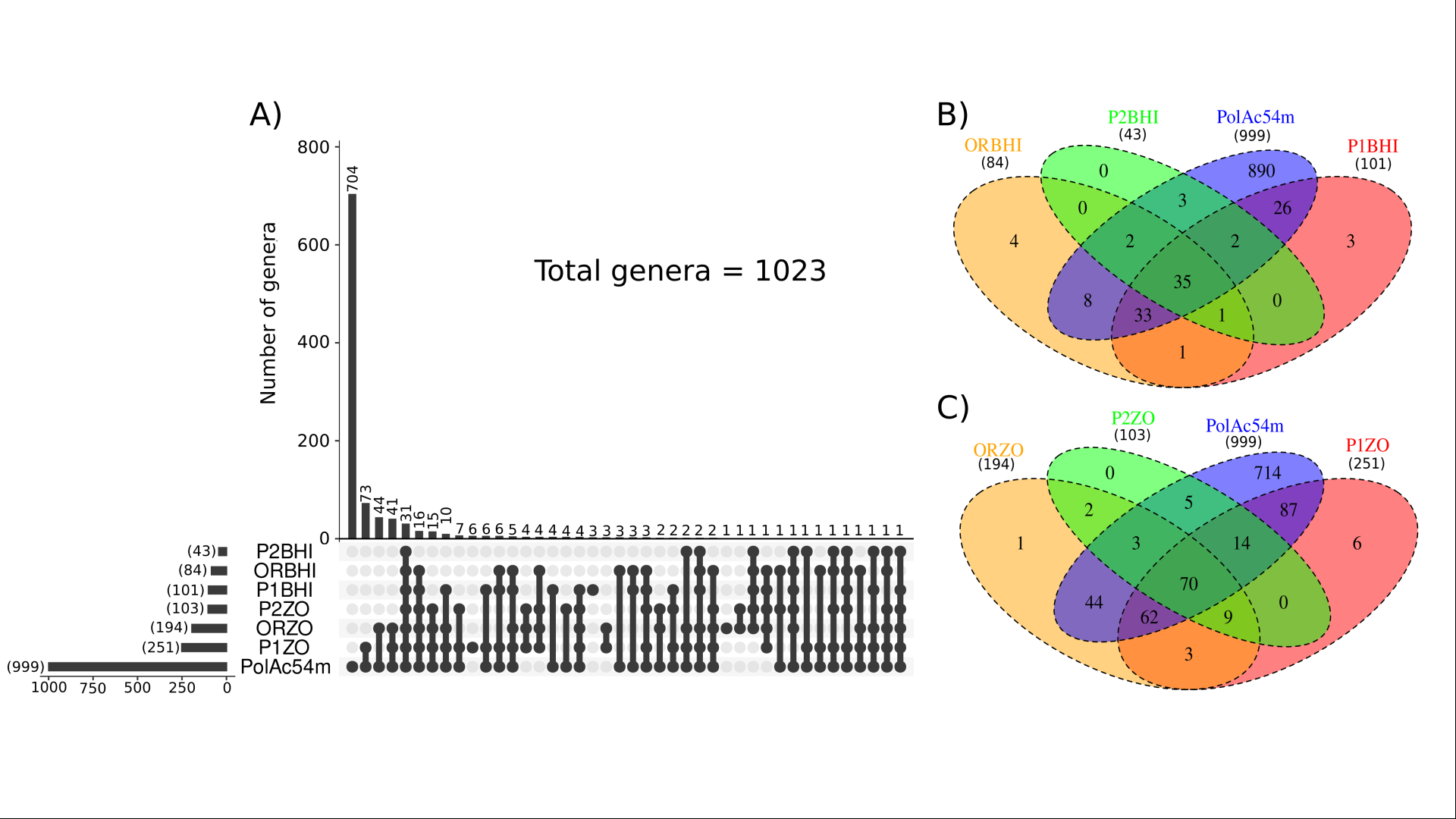


**Fig. A4.** BGCs detected in the 6 metagenomes by antiSMASH. Gene clusters are arranged top to bottom by the abundance of BGC-type


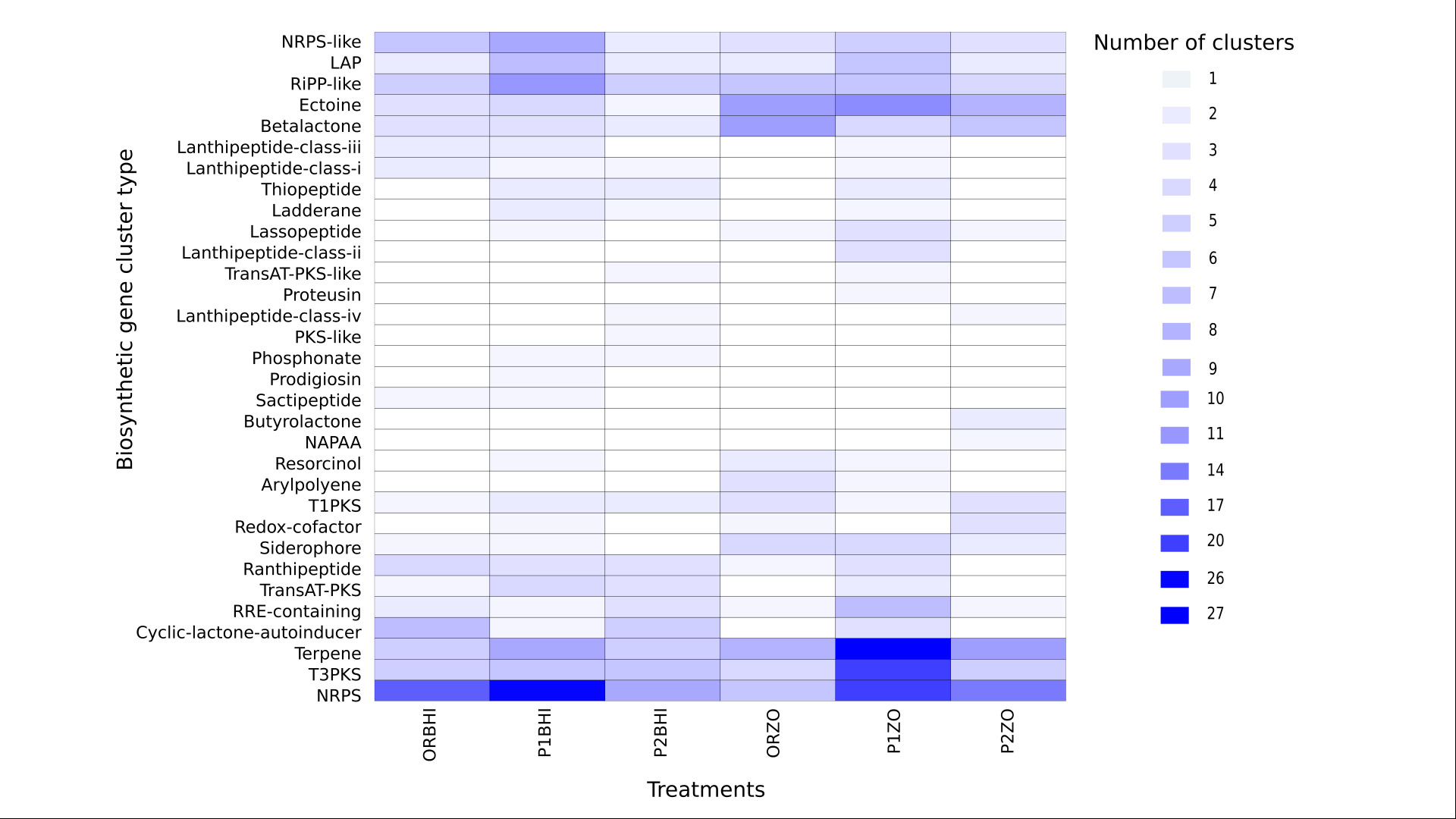


**Fig. A5.** Mapping of the reads of the environment sample against the contigs of the BGCs detected in the 6 metagenomes (a) and 35 high-quality MAGs (b) by antiSMASH. Gene clusters are arranged from top to bottom with their mean coverage.


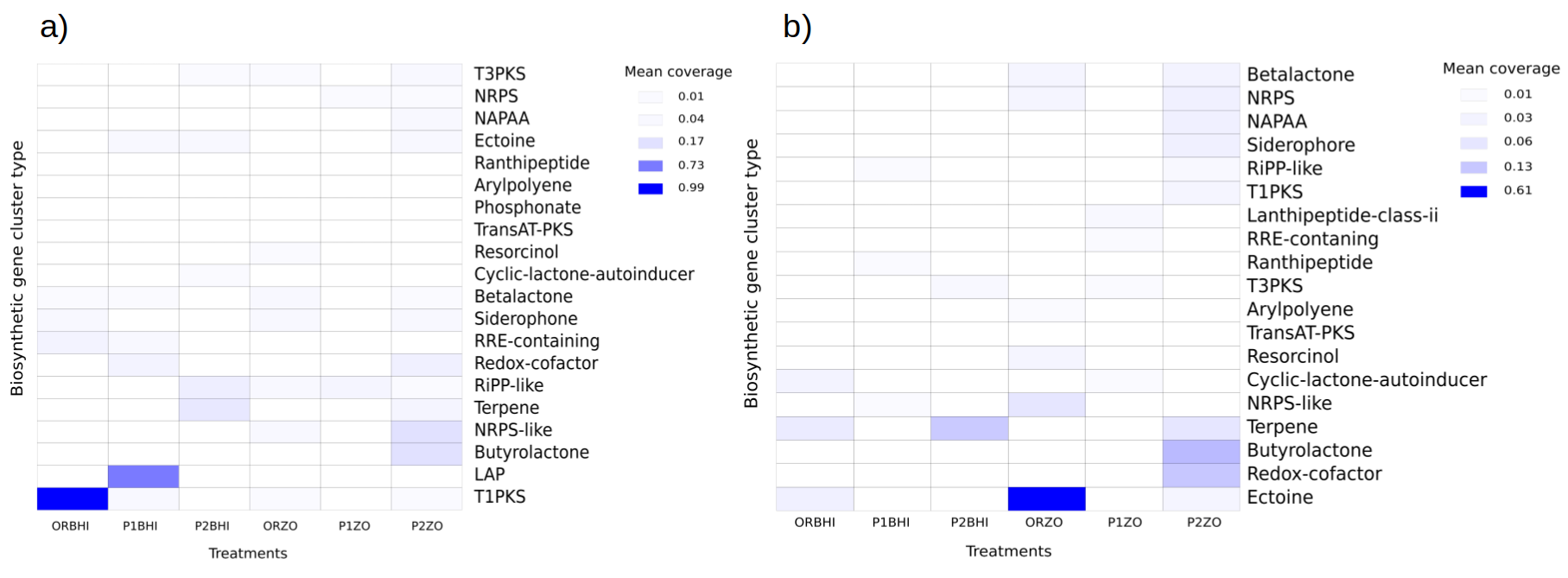


Fig.A6. Microbial community structure in the environmental metagenome (PolAc54M) and Coastal sinkhole (Zapote) of our previous study (Suarez-Moo et al. 2022). A) Venn diagrams showing the shared taxa (from phylum to genus) between the PolAc54M and Zapote sediments. The percentage is shown in parentheses. B) Relative abundance of taxa (from phylum to genus) based on 16S rRNA gene fragments of Illumina shotgun sequencing and 16S rRNA metabarcoding reads.


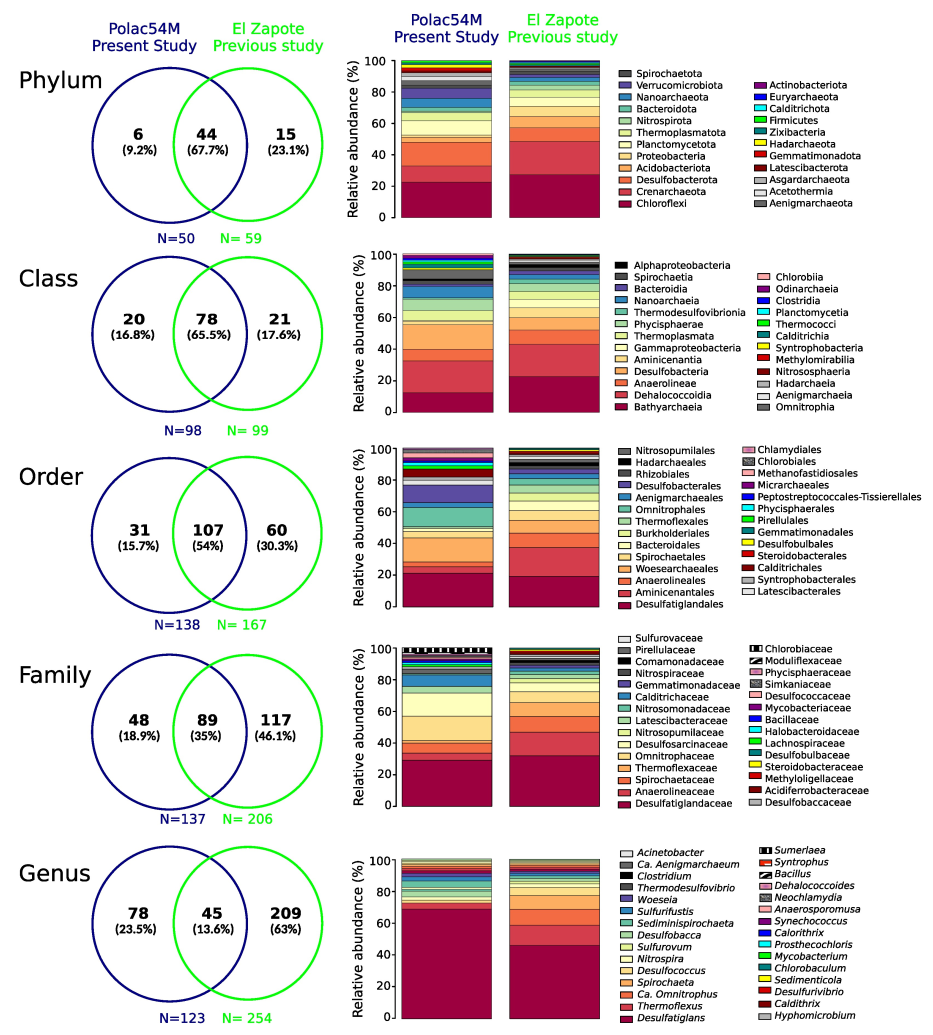


**Fig. A7.** Detailed annotation of BGCs from the 5 metabolite secondaries with the most similarity with known clusters from the MiBIG database; associated species are shown.


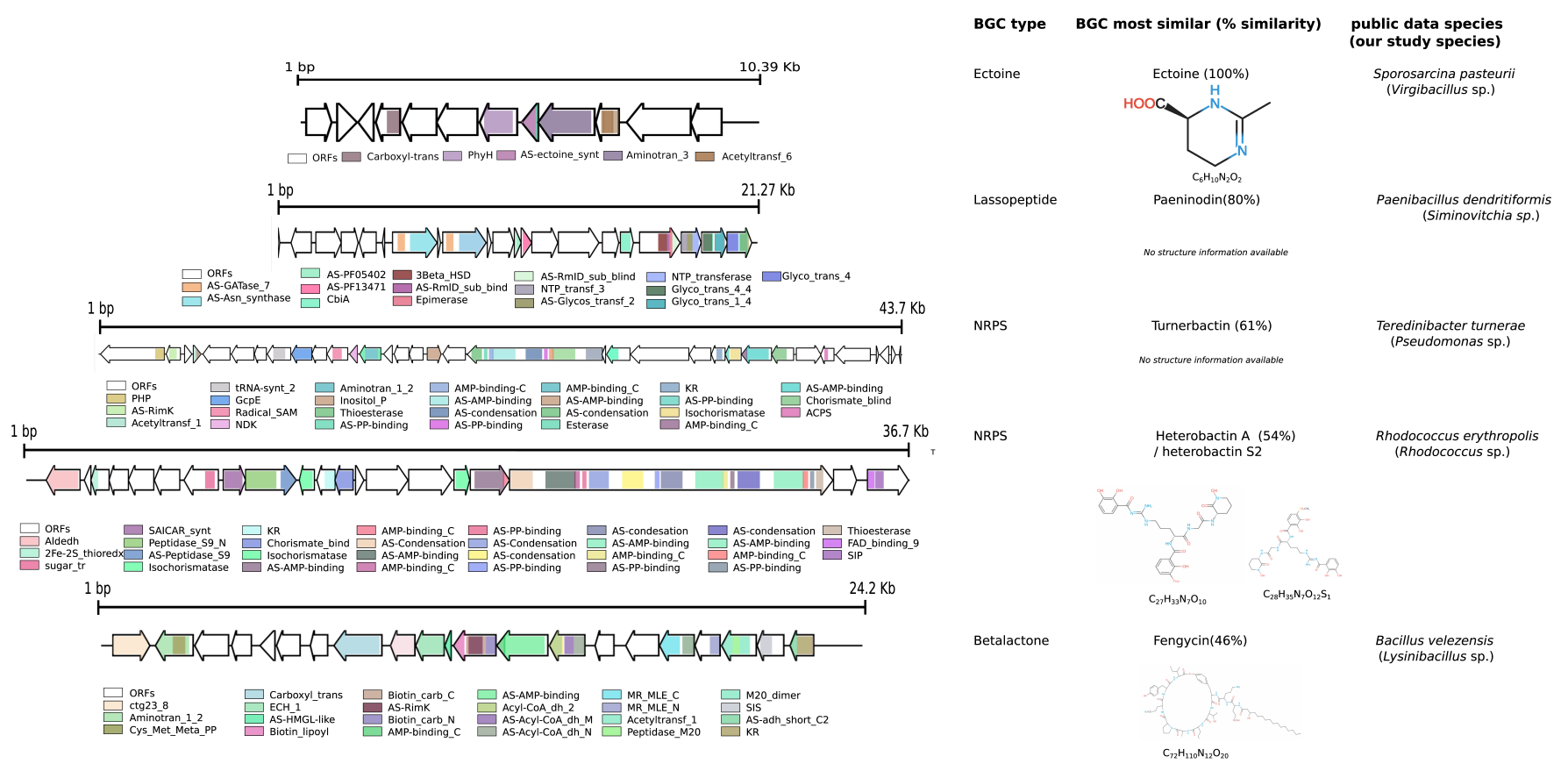


**Fig. A8.** Number of BGCs detected in the high-quality MAGs by (a) AntiSMASH, (b) NaPDoS, and (c) Bagel4. In parenthesis, total MAGs found for each treatment.


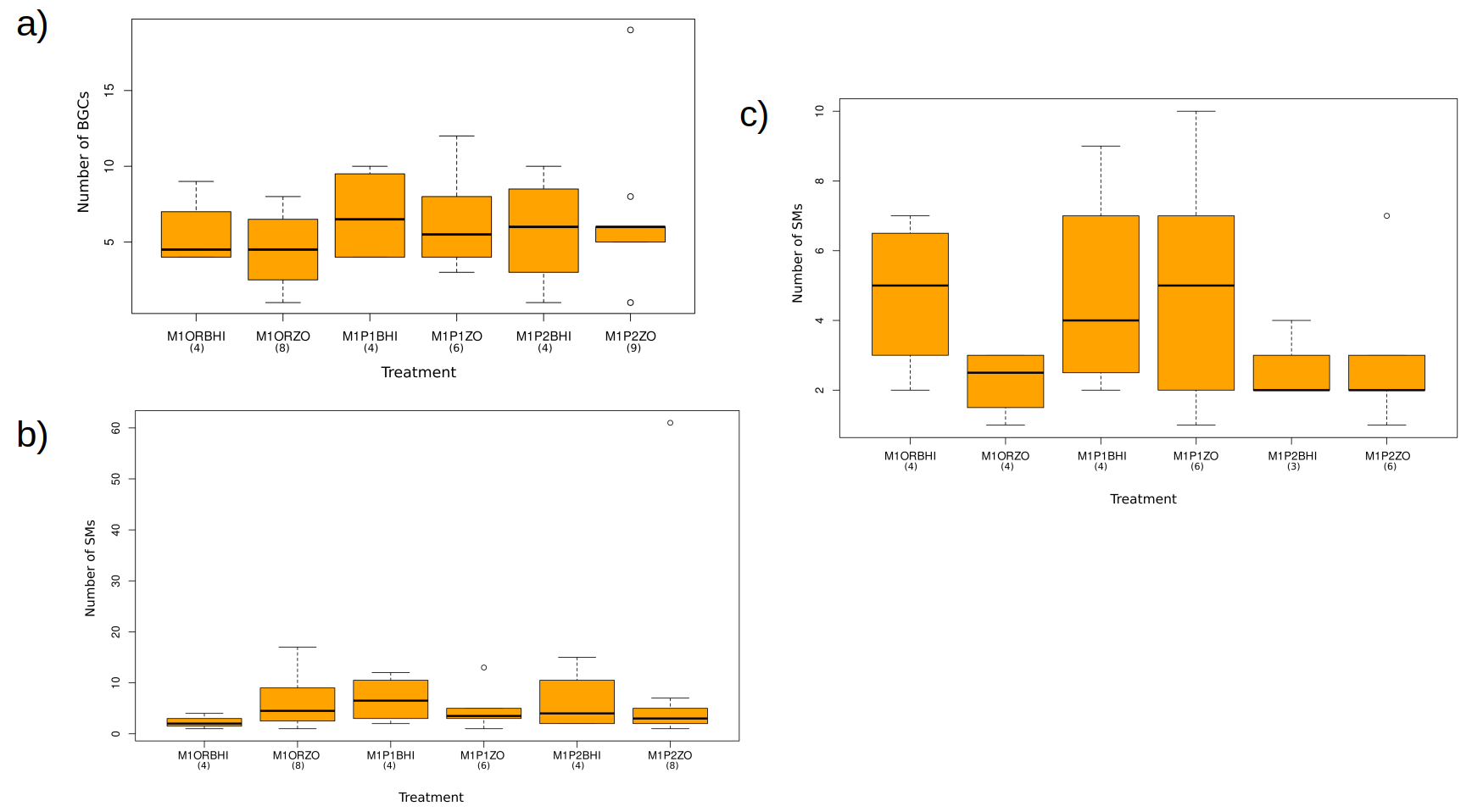

Supplement: Supplementary file 1 — Supporting information. [file MBO3-13-e1407-s002.docx]
